# Network Pharmacology Analysis of Traditional Chinese Medicine Formula *Xiao Ke Yin Shui* Treating Type 2 Diabetes Mellitus

**DOI:** 10.1155/2019/4202563

**Published:** 2019-09-08

**Authors:** Jiewen Zhou, Qiuyan Wang, Zhinan Xiang, Qilin Tong, Jun Pan, Luosheng Wan, Jiachun Chen

**Affiliations:** Hubei Key Laboratory of Natural Medicinal Chemistry and Resource Evaluation, School of Pharmacy, Huazhong University of Science and Technology, Hangkong Road 13#, Wuhan 430030, China

## Abstract

*Xiao Ke Yin Shui* (XKYS) formula is a traditional Chinese medicine formula treating type 2 diabetes mellitus (T2DM). XKYS formula consists of four herbs, i.e., Coptidis rhizoma, Liriopes radix, bitter melon, and Cassiae semen. Herein, the chemical profiles of four herb extracts were investigated, and further analysis of the underlying mechanism of XKYS formula treating T2DM was performed using network pharmacology. The main components were selected for our network-based research. Targets of XKYS formula were mainly collected from two databases, SwissTargetPrediction and Traditional Chinese Medicine Systems Pharmacology Database and Analysis Platform (TCMSP), and the text-mining method was also implemented. T2DM relating genes and therapeutic targets were collected from five databases. Subsequently, STRING and Cytoscape were employed for the analysis of protein-protein interaction (PPI) networks. Functional annotation and pathway analysis were conducted to investigate the functions and relating pathways of target genes. The content of 12 compounds in the herb extracts was determined. With the analysis of PPI networks, a total of 76 genes were found to be important nodes and could be defined as the main target genes regulated by XKYS formula in the treatment of T2DM and its complications. Components in XKYS formula mainly regulate proteins including protein kinase B (Akt), phosphatidylinositol 3-kinase (PI3K), insulin receptor substrate (IRS), and tumor necrosis factor (TNF). XKYS formula exerts therapeutic effects in a synergetic manner and exhibits antidiabetic effect mainly via reducing insulin resistance. These findings could be guidelines in the further investigation of this formula.

## 1. Introduction

Diabetes mellitus (DM) is now recognized as a complex metabolic disorder, and type 2 diabetes mellitus (T2DM) is the most common type of diabetes [1]. Nowadays, different kinds of therapies are applied in the treatment of diabetes, including insulin and other oral medications. Such therapies may be promising in glycemic control but could also cause side-effects like hypoglycemia or gastrointestinal dysfunction [2]. Thus, more and more people have turned their attention to herbal medicine or diet-based therapies, seeking for safer and more cost-effective complementary medicine for T2DM [3–6].

Traditional Chinese medicine (TCM) is a rich resource, possessing data that can still be hints for the development of new drugs. The focus of the current study, *Xiao Ke Yin Shui* (XKYS) formula, is recorded in *Bencaogangmu* (compendium of Materia Medica). XKYS formula contains four herbs, namely, Coptidis rhizoma (dried rhizomes of *Coptis chinensis* Franch.), Liriopes radix (the dried tuberous roots of *Liriope spicata* (Thunb.) Lour. var. *prolifera* Y. T. Ma), bitter melon (the immature fresh fruits of *Momordica charantia* L.), and Cassiae semen (the dried seeds of *Cassia obtusifolia* L.). All these herbs are widely used in clinical practice treating diabetes [7].

Several ingredients in these four herbs are generally accepted as the main bioactive components in the treatment of T2DM and its complications, i.e., alkaloids in Coptidis rhizoma, polysaccharides in Liriopes radix, triterpenoids and polysaccharides in bitter melon, and anthraquinone and naphthopyrone in Cassiae semen [8–11]. Thereby, herb extracts were prepared aiming to yield bioactive fractions containing ingredients mentioned above. The mixture of these herb extracts may be a satisfying alternative for the treatment of T2DM.

In XKYS formula, a single component may be responsible for the therapeutic effect through different pathways, and some components may act on the same targets as well. For example, isoquinoline alkaloids from Coptidis rhizoma like berberine are generally recognized as activators of adenosine 5′-monophosphate- (AMP-) activated protein kinase (AMPK) but could also exert therapeutic effects on T2DM through inhibition of protein tyrosine phosphatase 1B (PTP1B) and peroxisome proliferator-activated receptor gamma (PPAR*γ*) [12–14]. Polysaccharides of Liriopes radix and extracts of Cassiae semen could ameliorate glycemic control through activation of the phosphatidylinositol 3-kinase/protein kinase B (PI3K/Akt) signaling pathway [15, 16]. Triterpenoids in bitter melon are proved to be AMPK activators while polysaccharides are inhibitors of PPAR*γ* [17, 18]. These findings, however, could not fully explain the synergetic effects of XKYS formula in the treatment of T2DM and its complications.

Nowadays, researchers have been aware that the “one key, one lock” mode is insufficient to decipher the drug actions, especially in those complex diseases. Network pharmacology, however, analyzing drugs and drug targets in a systemic manner may provide us with novel insights into drug actions [19]. The key ideas of network pharmacology share much with the basic disciplines of TCM, making it a useful tool in the research of TCM [20]. In addition, rapid development of biomedical big data, like TCMSP (Traditional Chinese Medicine Systems Pharmacology Database and Analysis Platform), has facilitated such research [21]. Thus, a network-based pharmacological analysis could provide us with a comprehensive understanding towards the significance of each component, target, and pathway.

In brief, this study provided chemical profiles for the extracts of four herbs. And, network pharmacology analysis was applied to understand the underlying mechanisms of XKYS formula in the treatment of T2DM and its complications.

## 2. Materials and Methods

### 2.1. Materials and Reagent

Coptidis rhizoma, Liriopes radix, fresh bitter melon, and Cassiae semen were authenticated by Professor Jiachun Chen (School of Pharmacy, Huazhong University of Science and Technology, or HUST). Voucher specimens of these herbs were deposited in Hubei Key Laboratory of Natural Medicinal Chemistry and Resource Evaluation, School of Pharmacy, HUST.

The four herb extracts were total alkaloids of Coptidis rhizoma (TACR), Liriopes radix polysaccharides (LRP), bitter melon extract (BME), and Cassiae semen extract (CSE), respectively. Detailed preparation methods for the extracts were reported in Supplementary Materials (Part 1).

Acetonitrile (CH_3_CN) was purchased from Sigma Aldrich (USA). Water was deionized water. Hydrochloric acid, methanol, phosphate buffer, anthrone, sulfuric acid, fructose, glucose, and formic acid (HCOOH) were purchased from Sinopharm Chemical Reagent Co., Ltd. (Shanghai, China).

HPLC analysis of TACR, BME, and CSE was performed on the Agilent 1260 system with Agilent TC-C18 (250 mm × 4.6 mm, 5 *μ*m) columns. HPLC analysis of LRP was performed on the Hitachi L-2130 system with an Agilent TC-C18 (250 mm × 4.6 mm, 5 *μ*m) column. Content of total carbohydrate was determined on an ultraviolet-visible spectrophotometer, UV-1750 (Shimazu, Japan).

Epiberberine, coptisine, palmatine, berberine, and aurantio-obtusin were purchased from National Institute for Food and Drug Control. Cassiaside, rubrofusarin-6-O-*β*-D-gentiobioside, glucoaurantio-obtusin, and cassiaside C were purchased from Chengdu MUST Bio-technology Co. Ltd. (Sichuan, China). Momordicoside L, 7*β*,25-dihydrocucurbita-5,23(*E*)-dien-19-al-3-O-*β*-D-allopyranoside, and momordicoside F_2_ were self-prepared (Supplementary Materials, Part 2, Figures [Supplementary-material supplementary-material-1] and [Supplementary-material supplementary-material-1]). The purity of each standard was >98%.

### 2.2. Analysis of Four Herb Extracts

The analysis of TACR was conducted using the reported method [22].

The total carbohydrate content of LRP was examined using anthrone-sulfuric acid method according to the previous report [23].

Monosaccharide composition analysis of LRP was performed using the HPLC method after derivation with 1-pheny-3-methyl-5-pyrazolone (PMP), and the detailed method was reported in Supplementary Materials (Part 3) [24]. The mobile phase was 20 : 80 (v : v) CH_3_CN-H_3_PO_4_ buffer (pH 7.0). The flow rate was 1.0 ml/min. The injection volume was 20 *μ*l. Column temperature was held constant at 25°C. The UV detection wavelength was 250 nm.

The total carbohydrate content of BME was examined using anthrone-sulfuric acid method according to the previous report [23].

HPLC analysis of BME was conducted with the mobile phase consisting of CH_3_CN (A) and water (B). The elution program was 0 min 20%A, 10 min 30%A, 30 min 50%A, and 50 min 70%A. The flow rate was 1.0 ml/min. Column temperature was held constant at 25°C. The detector was evaporative light scattering detector (ELSD), with the evaporator temperature at 60°C and nebulizer temperature at 40°C. The flow rate of nitrogen was 1.6 l/min. The standard (10, 20 *μ*l) and sample (20 *μ*l) dissolved in 70% methanol were subjected to HPLC analysis.

HPLC analysis of CSE was carried out according to the previous report [25].

### 2.3. Construction of Protein-Protein Interaction (PPI) Networks of XKYS Formula Treating T2DM and Its Complications

To identify the corresponding targets of the main components of XKYS formula, several approaches combined with chemometric method, information integration, and data-mining were implemented. These components, except polysaccharides, were submitted to TCMSP and SwissTargetPrediction server to find component-target interaction information. TCMSP mainly predicts drug-target interactions with random forest (RF) and support vector machine (SVM) method, while SwissTargetPrediction is a 2D/3D similarity measurement of small molecules [26, 27]. All components, as well as the aglycon of the glycosides, were also submitted to PubMed, SciFinder, and Google Scholar for the text-mining of component-target information. And all targets were submitted to the UniProt database (http://www.uniprot.org) for validation of their gene names. Hereby, a target gene list of XKYS was obtained through *in silico* investigation.

Another gene list relating to T2DM was established after screening of five databases, including DrugBank database (http://www.drugbank.ca/), Online Mendelian Inheritance in Man (OMIM, http://www.omim.org/), Kyoto Encyclopedia of Genes and Genomes (KEGG, https://www.kegg.jp), Therapeutic Target Database (TTD, https://db.idrblab.org/ttd/), and Text-mined Hypertension, Obesity, and Diabetes Candidate Genes database (T-HOD, http://bws.iis.sinica.edu.tw/THOD/). And all genes and targets were submitted to the UniProt database for validation of their gene names.

Two gene lists were submitted to STRING. The STRING server generated a “Combined Score” ranged from 0 to 1 for each interaction. The higher the score is, the greater the confidence of the interaction. In STRING, an interaction >0.4 means “medium confidence” and >0.7 means “high confidence” [28].

The obtained PPI networks were intersected using Cytoscape 3.2.1, and interactions with score >0.7 were collected, generating a new PPI network, i.e., the PPI network of XKYS formula treating T2DM and its complications.

### 2.4. Gene Ontology (GO) Functional Annotation and KEGG Pathway Analysis

To elucidate the function of the targets and their role in signaling transduction, the Database for Annotation, Visualization and Integrated Discovery (DAVID) was used to analyze the GO function and KEGG pathway of the main target genes of XKYS formula in the treatment of T2DM and its complications. The biological process, cellular component, molecular function, and the pathway involved were also described.

### 2.5. Component-Target-Pathway Network Construction

The network model of component-target-pathway was established using Cytoscape 3.2.1. In this network, nodes represent components (C), targets (T), or pathways (P), and edges represent the interaction of C-T or T-P. Based on the results of KEGG enrichment analysis and C-T database, the C-T-P interactions were shown to provide an overview on the mechanisms of XKYS formula in the treatment of T2DM and its complications.

## 3. Results

### 3.1. Determination of Main Component Contents of Four Herb Extracts

**Figure 1:**
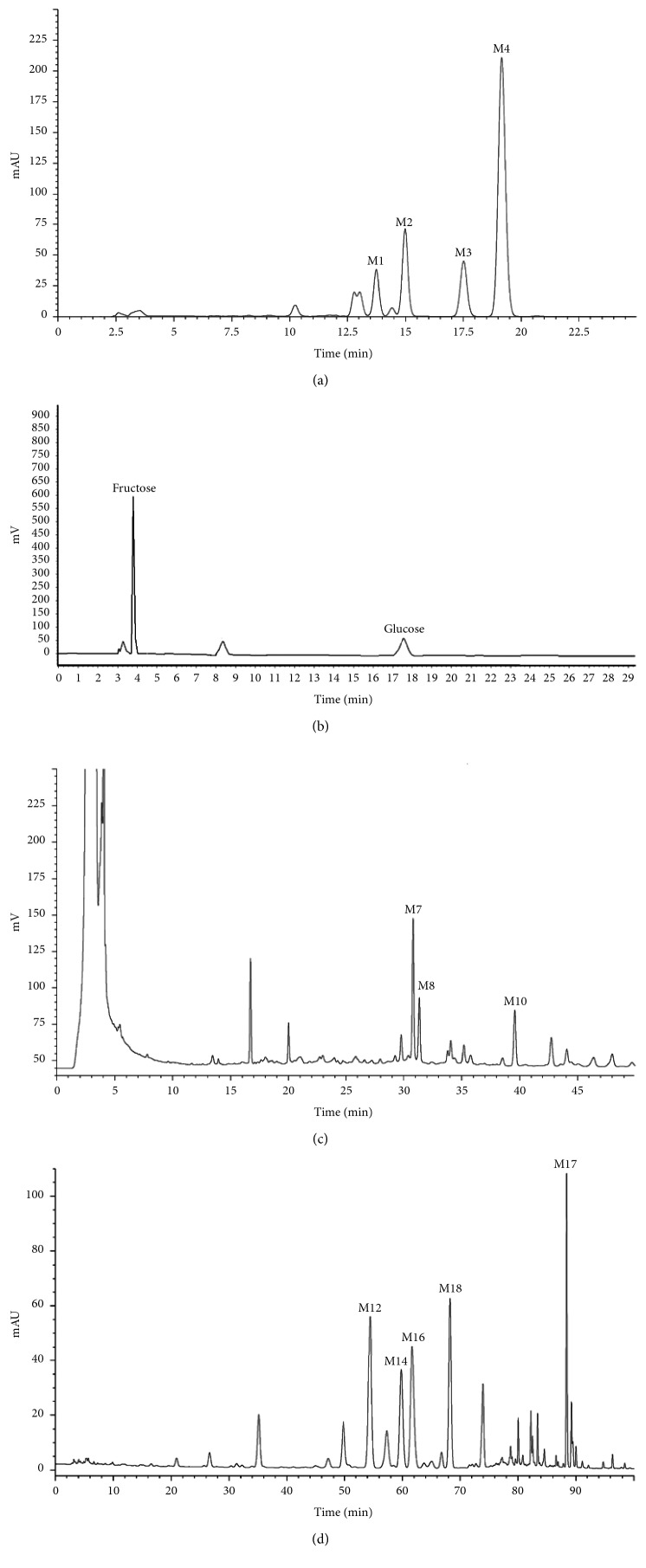
HPLC spectra of four herb extracts. (a) TACR: **M1**, epiberberine; **M2**, coptisine; **M3**, palmatine; **M4**, berberine. (b) Monosaccharide composition of LRP. Fructose : glucose = 20.7 : 1. (c) BME: **M7**, momordicoside L; **M8**, 7*β*,25-dihydrocucurbita-5,23(*E*)-dien-19-al-3-O-*β*-D-allopyranoside; **M10**, momordicoside F_2_. (d) CSE: **M12**, cassiaside; **M14**, rubrofusarin-6-O-*β*-D-gentiobioside, **M16**, glucoaurantio-obtusin; **M17**, aurantio-obtusin; **M18**, cassiaside C.

Four isoquinoline alkaloids, three cucurbitane-type triterpenoids, two anthraquinones, and three naphthopyrones were determined using HPLC. Results of the HPLC analysis are shown in Table 1 and Figure 1, and the structures were also reported (Table 2). The total content of carbohydrate in LRP was determined to be 96.5% while BME 10.8%. The composition of LRP was also determined as fructose : glucose, 20.7 : 1.

### 3.2. Screening of Main Components from Four Herb Extracts and Construction of PPI Networks

**Figure 2:**
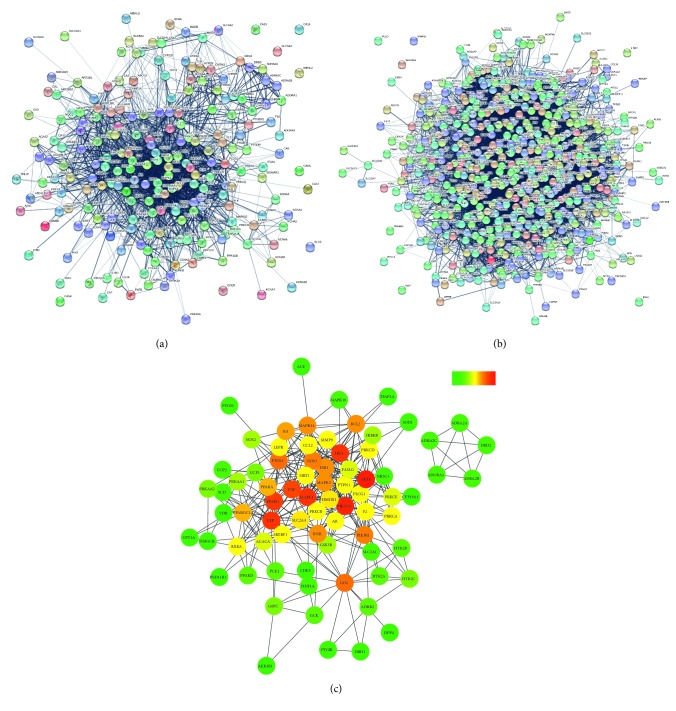
Protein-protein interaction networks. The mapping of PPI network was generated by the STRING server. (a) PPI network of genes regulated by XKYS formula (217 nodes, 2070 edges). (b) PPI network of genes relating to the pathophysiology of T2DM (602 nodes, 11595 edges). (c) 76 main target genes regulated by XKYS formula in the treatment of T2DM and its complications. This network contains 76 nodes and 333 edges. As shown in the color bar, nodes in red could be considered as important and nodes in green are less important in this network. The degree value of each node in Figure 2(c) is presented in Table 3.

The screening of 19 components (Table 2) in XKYS had led to the acquisition of 216 targets, while 602 genes relating to the pathophysiology of T2DM were also collected (Supplementary Materials, Tables [Supplementary-material supplementary-material-1] and [Supplementary-material supplementary-material-1]). All the gene names in two lists were uploaded to STRING, respectively. The XKYS PPI network consists of 216 proteins and 2070 interactions, whereas T2DM, 602 proteins and 11595 interactions (Figures 2(a) and 2(b)). Each interaction has a Combined Score >0.4. By intersecting these two networks, all interactions with high confidence (>0.7) were picked, generating a new PPI network.

The new network consists of 76 genes, representing the main target genes regulated by XKYS formula in the treatment of T2DM. In addition, among these genes, some genes like AKT1, mitogen-activated protein kinase 1 (MAPK1), tumor necrosis factor (TNF) were also found to be important genes involved in the progression of diabetic complications [29–31]. Thus, these genes could also be regarded as the main target genes regulated by XKYS formula in the treatment of T2DM and its complications (Figure 2(c), Table 3).

The new PPI network contains 76 nodes and 333 edges (Figure 2(c)). In this network, nodes represent targets, while edges, the interactions of proteins. And degree, a topological parameter describing the importance of a node, stands for the number of edges connecting to the node. The higher the degree, the more important the target in the network is.

As can be seen in Figure 2(c), important targets were painted red and located centrally in the network. AKT1, PI3KCG, MAPK1, LEP, IRS1, and PPARG were the top six genes regarding their degree.

### 3.3. GO Functional Annotation and KEGG Pathway Analysis

**Figure 3:**
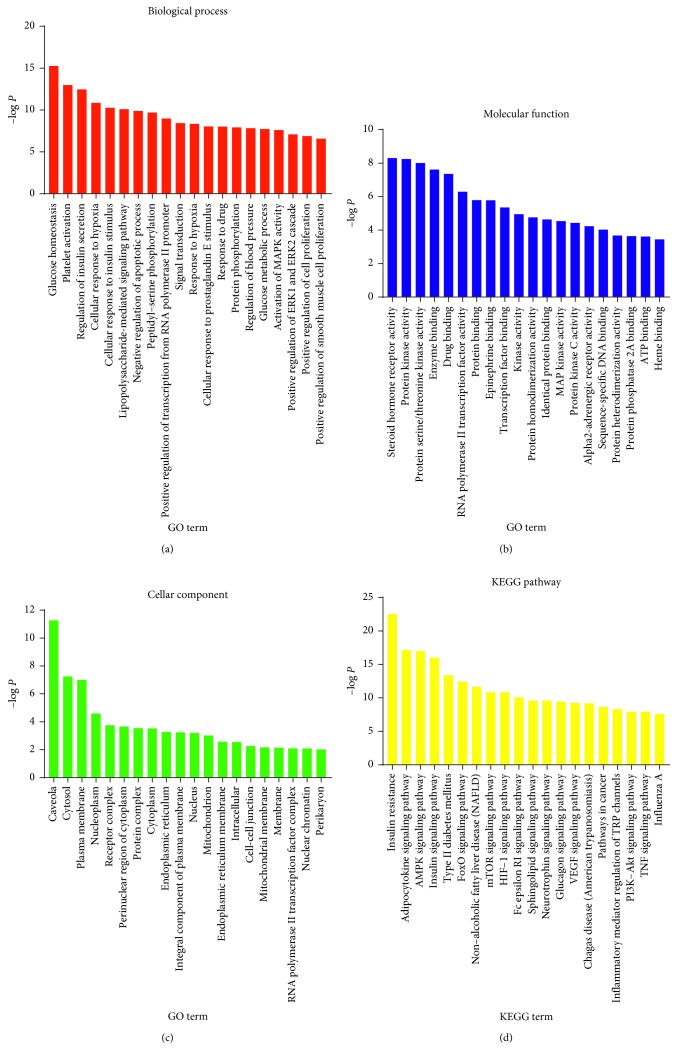
GO functional annotation and KEGG analysis of 76 target genes. Biological process (a), molecular function (b), cellar components (c), and KEGG pathways (d) were sorted according to −log *P* values (*P* < 0.01). *P* value was calculated using a modified Fisher exact test, which ranges from 0 to 1. *P* value = 0 represents perfect enrichment. For each diagram, the *x*-axis presents GO or KEGG terms, whereas the *y*-axis is the −log *P* value of each term.

GO functional annotation was performed on 76 target genes, and the top 20 GO terms (*P* < 0.01) were selected based on −log *P* values (Figure 3). GO enrichment analysis indicated that these 76 target genes are responsible for glucose homeostasis, platelet activation, regulation of insulin secretion, cellar response to hypoxia, and cellular response to insulin stimulus (Figure 3(a)). These biological processes are related to molecular functions including, steroid hormone receptor activity, protein kinase activity, protein serine/threonine (Ser/Thr) kinase activity, enzyme binding, and drug binding (Figure 3(b)). And, these processes occur mainly in caveola, cytosol, plasma membrane, nucleoplasm, and receptor complex (Figure 3(c)).

KEGG pathway analysis was conducted for further exploration of these targets as shown in Figure 3(d). These targets were highly enriched in insulin resistance (IR), insulin pathway, adipocytokine pathway, AMPK pathway, T2DM, forkhead box protein O (FoxO) pathway, nonalcoholic fatty liver disease (NAFLD), mammalian target of rapamycin (mTOR) pathway, hypoxia-inducible factor 1 (HIF-1) pathway, glucagon pathway, and so on. These pathways are highly relevant to the development of T2DM and its complications, and XKYS formula may exert therapeutic effects through pathways mentioned above.

### 3.4. Composition-Target-Pathway Network Construction

**Figure 4:**
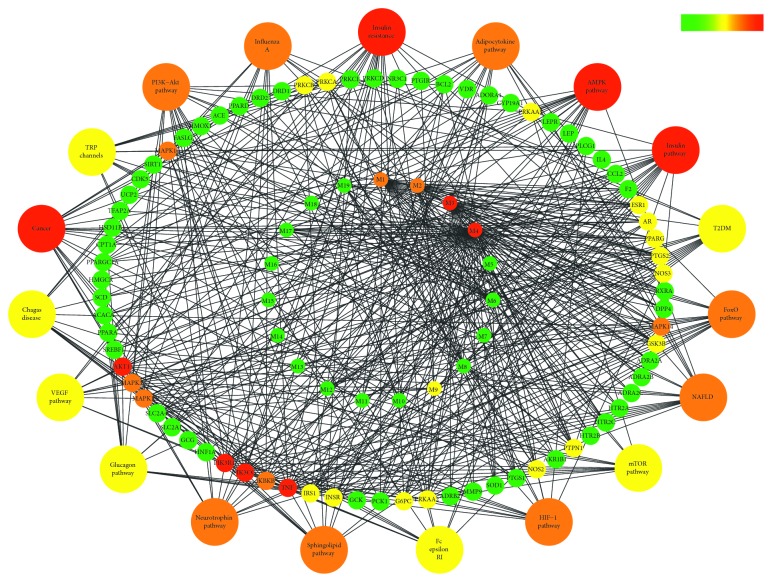
Component-target-pathway network of XKYS formula in the treatment of T2DM and its complications. Nodes in red are considered as important, and the green nodes are considered as less important. The degree of each node was presented in Supplementary Materials [Supplementary-material supplementary-material-1].

A total of 76 genes were defined as the main target genes regulated by XKYS formula in the treatment of T2DM and its complications. These genes were kept, and other entries were omitted in the database of C-T. The combination of C-T and T-P databases had led to a C-T-P network (Figure 4), providing us with an overview on the therapeutic effects of XKYS formula. In addition, the degree of each node is presented in Supplementary Materials [Supplementary-material supplementary-material-1].

The inner cycle represents the main components of XKYS formula. Nodes painted in red were key components interacting with a larger number of targets. Among all of these 19 components, alkaloids from Coptidis rhizoma were regarded as the key components in the treatment of T2DM and its complications.

The middle cycle represents the main target genes regulated by XKYS formula. When painted in red, the corresponding targets are regulated by more components and participate in more pathways. It could be observed that PI3KR1, PI3KCG, AKT, and TNF are key genes regulated by XKYS formula. This result agreed with that obtained from PPI analysis.

The outer cycle represents the top 20 pathways enriched. Pathways containing the most target genes were in red. IR, AMPK pathway, insulin pathway, and pathway in cancer are the key pathways. This result was familiar to that obtained from KEGG pathway analysis.

## 4. Discussion

TCM formulae are usually hard to decipher due to its mode of action, namely, “network target, multicomponents” [20]. T2DM is also a complex metabolic disorder with changes in various pathways. XKYS formula and T2DM can be considered as two networks and explained with the help of network pharmacology.

According to C-T-P network, alkaloids from Coptidis rhizoma are the main components in XKYS formula that exert antidiabetic effects. Results also indicated that around 64% of the content in TACR could be determined using the HPLC method. Among these isoquinoline alkaloids determined, berberine accounted for ∼35% of the fraction and was also proved to be a key node in the C-T-P network. Berberine has a wide range of biological activities, among which anti-hyperglycemic and anti-hyperlipidemia are two major benefits in the treatment of T2DM [8]. Berberine could activate AMPK via inhibition of mitochondrion respiratory complex I instead of the regulation of calcium/calmodulin-dependent protein kinase kinase beta (CaMKK*β*), leading to the reduction of IR and amelioration of lipid metabolism [13, 32]. Momordicoside L and its analogue were detected in BME and interesting enough, and the agylcon of Momordicoside L could activate AMPK through CaMKK*β*, which was distinct from the mechanism of berberine [17]. This could be a good evidence of synergetic effect presented by this formula.

LRP are highly enriched according to its total content of carbohydrate. Our previous studies had found that LRP could upregulate the insulin signaling pathway and exert therapeutic effects in diabetic rodents through activation of PI3K/Akt signaling pathway [15, 23]. Aurantio-obtusin, along with its glycosides, accounted for ∼6.8% of CSE and was shown to be an important component in the C-T-P network. Previous reports had indicated that aurantio-obtusin may offer therapeutic effects in hypertension through the PI3K/Akt-eNOS (endothelial nitric oxide synthase) pathway, implying a possible mechanism in the treatment of diabetic complications of XKYS formula [33].

As can be seen in the C-T-P network, AKT1, PI3KCG, PI3KR1, and TNF were key genes regulated by XKYS formula in the treatment of T2DM and its complications. Proteins expressed by genes AKT1, PI3KCG, and PI3KR1 were all important members in the signaling transduction of PI3K/Akt. This signaling pathway is of great significance in the progression of T2DM due to its role in glucose metabolism [34]. The PI3K/Akt signaling pathway is also responsible for the regulation of the signaling pathway like MAPK, FoxO, and nuclear factor kappa B (NF-*κ*B). These pathways play important roles in the regulation of protein synthesis, cell survival, differentiation, proliferation, and apoptosis and are highly related to the proliferation and regeneration of islet *β*-cells [29].

Tumor necrosis factor (TNF) is a cytokine that could be secreted by macrophages and adipose cells. It can induce IR by downregulation of the activity of PI3K/Akt signaling pathway. It has also been reported that MAPK and NF-*κ*B signaling pathways are stimulated by TNF and further regulates inflammatory response, oxidative stress, and apoptosis [31].

As shown in KEGG pathway analysis, 23 out of 76 targets were found to participate in IR, ranking no. 1 according to its −log *P* value. Other pathways on the KEGG list were adipocytokine pathway, AMPK pathway, insulin pathway, T2DM, FoxO pathway, NAFLD, etc. And, this finding was further approved by the C-T-P network. All of these signaling pathways are all highly related to IR. Thus, we could make a preliminary inference from this result that the most important mechanism of XKYS formula in the treatment of T2DM may be reducing IR.

IR is a condition in which the target organs are insensitive to insulin. Long-term of unhealthy diet and lacking exercises could cause overweight or even obesity. Overweight or obese patients are in metabolic disorder states with excessive fat accumulation [35]. Some experts had put forward a hypothesis that obesity is a chronic condition of inflammation [36]. Adipocytokines like TNF and interleukin-6 (IL-6) could inhibit the binding of insulin receptor (InsR) and insulin receptor subunit (IRS). IRS would be degraded under such conditions. In addition, inflammation response and oxidative stress interact as both cause and effect, causing the damage of islet *β*-cells and diabetic microangiopathy and then leading to the onset of T2DM and its complications [37]. Moreover, the activity of AMPK in skeletal muscles and liver reduces in IR conditions, causing decreased oxidation of free fatty acids and a lower intake of glucose, which in turn deteriorating glycemic control [38]. The FoxO pathway is one of those key factors in the transition from IR to the damage of islet *β*-cells. The dysfunction of *β*-cells is caused by various factors, including oxidative stress and inflammatory response. The FoxO pathway is highly related to the risk factors mentioned above [39].

In addition, the pathway in cancer was also an important pathway enriched in the C-T-P network (Figure 4). For one thing, diabetes is a risk factor for carcinogenesis due to hyperinsulinemia, hyperglycemia, and fat-induced chronic inflammation [40]. For another, components in XKYS formula like isoquinoline alkaloids and cucurbitane-type triterpenoids were reported to possess antitumor activities through pathways participating in the pathophysiology of both diabetes and cancer [41, 42]. Thus, such correlation was also presented in C-T-P network.

It should also be noted that the PI3K/Akt signaling pathway is also regarded as a key regulator in the development of diabetic complications [29]. And, among those pathways enriched, the hypoxia-inducible factor 1 (HIF-1) pathway and vascular endothelial growth factor (VEGF) pathway are considered to be important factors involved in the development of diabetic complications [43, 44].

The network-based pharmacological analysis has shown that XKYS formula could regulate the glucose and lipid metabolism and alleviate IR mainly through insulin, AMPK, adipocytokine, and FoxO pathway. And, this formula could also be used in the treatment of diabetic complications due to its effect on PI3K/Akt, HIF-1, and VEGF pathways.

This study provided an overview on the antidiabetic effects of XKYS formula in a holistic manner. However, in vivo and in vitro experiments are required to offer more information about the mechanisms of XKYS formula.

## 5. Conclusion

Components in XKYS formula mainly regulate proteins including Akt, PI3K, IRS, and TNF. XKYS formula exerts therapeutic effects in a synergetic manner and exhibits antidiabetic effect mainly via reducing IR. These findings could be guidelines in the further investigation of this formula.

## Figures and Tables

**Table 1:** Content of components in herb extracts determined using HPLC.

Extracts	Constituents	Content (mg/g)
TACR	Epiberberine	55.0
	Coptisine	135.1
	Palmatine	99.7
	Berberine	354.7

BME	Momordicoside L	1.9
	7*β*,25-Dihydrocucurbita-5,23(*E*)-dien-19-al3-O-*β*-D-allopyranoside	1.0
	Momordicoside F_2_	1.9

CSE	Cassiaside	40.0
	Rubrofusarin 6-O-*β*-D-gentiobioside	32.0
	Glucoaurantio-obtusin	46.1
	Cassiaside C	27.4
	Aurantio-obtusin	22.2

**Table 2:** Structures of components in four herb extracts.

No.	Name	CAS No.	Molecular formula	Structure	Herb
**M1**	Epiberberine	6873-09-2	C_20_H_18_NO_4_	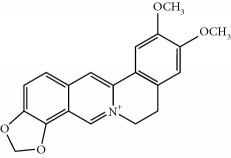	Coptidis rhizoma
**M2**	Coptisine	3486-66-6	C_19_H_14_NO_4_	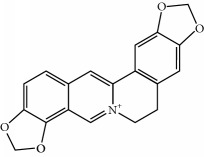	Coptidis rhizoma
**M3**	Palmatine	3486-67-7	C_21_H_22_NO_4_	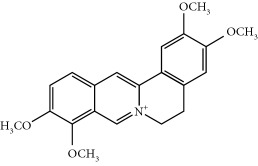	Coptidis rhizoma
**M4**	Berberine	2086-83-1	C_20_H_18_NO_4_	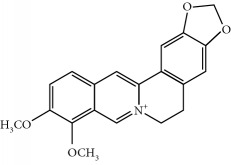	Coptidis rhizoma
**M5**	Liriopes radix polysaccharides	N/A	N/A	N/A	Liriopes radix
**M6**	Bitter melon polysaccharides	N/A	N/A	N/A	Bitter melon
**M7**	Momordicoside L	81348-83-6	C_36_H_58_O_9_	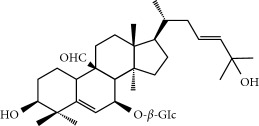	Bitter melon
**M8**	7*β*,25-Dihydrocucurbita-5,23(*E*)-dien-19-al-3-O-*β*-D-allopyranoside	912329-04-5	C_36_H_58_O_9_	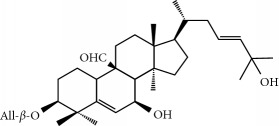	Bitter melon
**M9**	3,7,25-Trihydroxycucurbita-5,23-dien-19-al	85372-65-2	C_30_H_48_O_4_	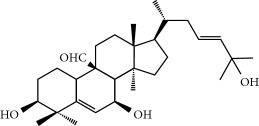	Bitter melon
**M10**	Momordicoside F_2_	81348-82-5	C_36_H_58_O_8_	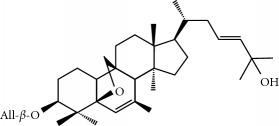	Bitter melon
**M11**	5*β*,19-Epoxycucurbita-6,23-diene-3*β*,25-diol	81910-41-0	C_30_H_48_O_3_	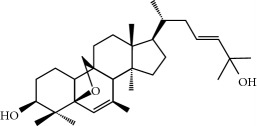	Bitter melon
**M12**	Cassiaside	13709-03-0	C_20_H_20_O_10_	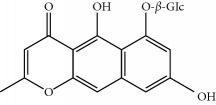	Cassiae semen
**M13**	Norrubrofusarin	3566-98-1	C_14_H_10_O_5_	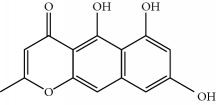	Cassiae semen
**M14**	Rubrofusarin 6-O-*β*-D-gentiobioside	24577-90-0	C_27_H_32_O_15_	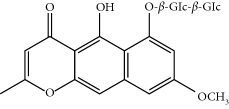	Cassiae semen
**M15**	Rubrofusarin	3567-00-8	C_15_H_12_O_5_	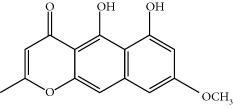	Cassiae semen
**M16**	Glucoaurantio-obtusin	129025-96-3	C_23_H_24_O_12_	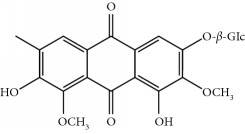	Cassiae semen
**M17**	Aurantio-obtusin	67979-25-3	C_17_H_14_O_7_	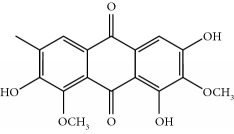	Cassiae semen
**M18**	Cassiaside C	119170-52-4	C_27_H_32_O_15_	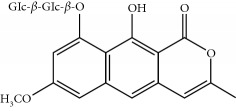	Cassiae semen
**M19**	Toralactone	41743-74-2	C_15_H_12_O_5_	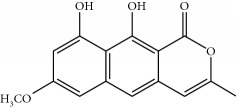	Cassiae semen

N/A = not applicable.

**Table 3:** Main targets regulated by XKYS formula in the treatment of T2DM and its complications.

Protein name	Gene name	Unipro ID	PPI network degree
RAC-alpha serine/threonine-protein kinase	AKT1	P31749	27
Phosphatidylinositol 4,5-bisphosphate 3-kinase catalytic subunit gamma isoform	PIK3CG	P48736	24
Insulin receptor substrate 1	IRS1	P35568	22
Leptin	LEP	P41159	22
Mitogen-activated protein kinase 1	MAPK1	P28482	22
Peroxisome proliferator activated receptor gamma	PPARG	P37231	21
Tumor necrosis factor	TNF	P01375	20
Glucagon	GCG	P01275	17
Prostaglandin G/H synthase 2	PTGS2	P35354	17
Nitric-oxide synthase, endothelial	NOS3	P29474	16
Phosphatidylinositol 3-kinase regulatory subunit alpha	PIK3R1	P27986	16
Apoptosis regulator Bcl-2	BCL2	P10415	15
Estrogen receptor	ESR1	P03372	15
Insulin receptor	INSR	P06213	15
Mitogen-activated protein kinase 14	MAPK14	Q16539	15
Mitogen-activated protein kinase 3	MAPK3	P27361	15
Interleukin-4	IL4	P05112	14
Peroxisome proliferator-activated receptor alpha	PPARA	Q07869	13
Peroxisome proliferator-activated receptor gamma coactivator 1-alpha	PPARGC1A	Q9UBK2	13
Heme oxygenase 1	HMOX1	P09601	11
Solute carrier family 2, facilitated glucose transporter member 4	SLC2A4	P14672	11
C-C motif chemokine 2	CCL2	P13500	10
Prothrombin	F2	P00734	10
Matrix metalloproteinase-9	MMP9	P14780	10
Protein kinase C delta type	PRKCD	Q05655	10
NAD-dependent protein deacetylase sirtuin-1	SIRT1	Q96EB6	10
Sterol regulatory element-binding protein 1	SREBF1	P36956	10
Androgen receptor	AR	P10275	9
Leptin receptor, LEP-R	LEPR	P48357	9
1-Phosphatidylinositol 4,5-bisphosphate phosphodiesterase gamma-1	PLCG1	P19174	9
Protein kinase C alpha type	PRKCA	P17252	9
Protein kinase C beta type	PRKCB	P05771	9
Tyrosine-protein phosphatase nonreceptor type 1	PTPN1	P18031	9
Retinoic acid receptor RXR-alpha	RXRA	P19793	9
Acetyl-CoA carboxylase 1	ACACA	Q13085	8
Tumor necrosis factor ligand superfamily member 6	FASLG	P48023	8
Protein kinase C epsilon type	PRKCE	Q02156	8
Glycogen synthase kinase-3 beta	GSK3B	P49841	7
Inhibitor of nuclear factor kappa-B kinase subunit beta	IKBKB	O14920	7
Nitric oxide synthase, inducible	NOS2	P35228	7
5′-AMP-activated protein kinase catalytic subunit alpha-1	PRKAA1	Q13131	7
Glucose-6-phosphatase	G6PC	P35575	6
5-Hydroxytryptamine receptor 2C (by homology)	HTR2C	P28335	6
5′-AMP-activated protein kinase catalytic subunit alpha-2	PRKAA2	P54646	6
Mitochondrial brown fat uncoupling protein 1	UCP1	P25874	6
Beta-2 adrenergic receptor	ADRB2	P07550	5
Glucokinase	GCK	P35557	5
5-Hydroxytryptamine receptor 2A (by homology)	HTR2A	P28223	5
5-Hydroxytryptamine receptor 2B	HTR2B	P41595	5
Phosphoenolpyruvate carboxykinase, cytosolic (GTP)	PCK1	P35558	5
Peroxisome proliferator-activated receptor delta	PPARD	Q03181	5
Adenosine receptor A1	ADORA1	P30542	4
Alpha-2A adrenergic receptor (by homology)	ADRA2A	P08913	4
Alpha-2B adrenergic receptor	ADRA2B	P18089	4
Alpha-2C adrenergic receptor	ADRA2C	P18825	4
D(2) dopamine receptor (by homology)	DRD2	P14416	4
Hepatocyte nuclear factor 1-alpha	HNF1A	P20823	4
Mitogen-activated protein kinase 10	MAPK10	P53779	4
Glucocorticoid receptor	NR3C1	P04150	4
Acyl-CoA desaturase	SCD	O00767	4
Solute carrier family 2, facilitated glucose transporter member 1	SLC2A1	P11166	4
Mitochondrial uncoupling protein 2	UCP2	P55851	4
Carnitine O-palmitoyltransferase 1, liver isoform	CPT1A	P50416	3
D(1A) dopamine receptor	DRD1	P21728	3
3-Hydroxy-3-methylglutaryl-coenzyme A reductase	HMGCR	P04035	3
Prostacyclin receptor	PTGIR	P43119	3
Superoxide dismutase (Cu-Zn)	SOD1	P00441	3
Aldose reductase	AKR1B1	P15121	2
Cyclin-dependent-like kinase 5	CDK5	Q00535	2
Aromatase	CYP19A1	P11511	2
Corticosteroid 11-beta-dehydrogenase isozyme 1	HSD11B1	P28845	2
Transcription factor AP-2-alpha	TFAP2A	P05549	2
Vitamin D3 receptor	VDR	P11473	2
Angiotensin-converting enzyme	ACE	P12821	1
Dipeptidyl peptidase 4	DPP4	P27487	1
Prostaglandin G/H synthase 1	PTGS1	P23219	1

## Data Availability

The data used to support the findings of this study are included within the article and the Supplementary Materials.

## Abbreviations

Akt: Protein kinase B
AMPK: Adenosine 5′-monophosphate (AMP) activated protein kinase
BME: Bitter melon extract
CaMKK*β*: Calcium/calmodulin-dependent protein kinase kinase beta
CSE: Cassiae Semen extract
DM: Diabetes mellitus
FoxO: Forkhead box protein O
GO: Gene ontology
HIF-1: Hypoxia-inducible factor 1
InsR: Insulin receptor
IR: Insulin resistance
IRS: Insulin receptor subunit
KEGG: Kyoto encyclopedia of genes and genomes
LRP: Liriopes radix polysaccharides
mTOR: Mammalian target of rapamycin
NAFLD: Nonalcoholic fatty liver disease
NF-*κ*B: Nuclear factor kappa B
PI3K: Phosphatidylinositol 3-kinase
PPAR*γ*: Peroxisome proliferator-activated receptor gamma
PPI: Protein-protein interaction
T2DM: Type 2 diabetes mellitus
TACR: Total alkaloids of Coptidis rhizoma
TCM: Traditional Chinese medicine
TCMSP: Traditional Chinese medicine systems pharmacology database and analysis platform
TNF: Tumor necrosis factor
VEGF: Vascular endothelial growth factor
XKYS formula: *Xiao Ke Yin Shui* formula.

## Supplementary Materials

Supplementary Materials Figure S1: structures of M7, M8, and M10. Figure S2: HPLC spectra of M7 (A), M8 (B), and M10 (C) and bitter melon extract (D). Table S1: *in silico* screening results of XKYS. Table S2: the gene list collected from online resources relating to T2DM. Table S3: information of each node in the component-target-pathway network.
