# Supplementary material for: Network Pharmacology Analysis of Traditional Chinese Medicine Formula Xiao Ke Yin Shui Treating Type 2 Diabetes Mellitus
Source: Evid Based Complement Alternat Med. 2019 Sep 8;2019:4202563. doi: 10.1155/2019/4202563 (PMC6754917; doi:10.1155/2019/4202563)
Supplement: Supplementary Materials — Figure S1: structures of M7, M8, and M10. Figure S2: HPLC spectra of M7 (A), M8 (B), and M10 (C) and bitter melon extract (D). Table S1: in silico screening results of XKYS. Table S2: the gene list collected from online resources relating to T2DM. Table S3: information of each node in the component-target-pathway network. [file 4202563.f1.docx]

**Supplementary Material**

**Evidence-Based Complementary and Alternative Medicine**

**Network Pharmacology Analysis of Traditional Chinese Medicine Formula *Xiao Ke Yin Shui* Treating Type 2 Diabetes Mellitus**

Jiewen Zhou, Qiuyan Wang, Zhinan Xiang, Qilin Tong, Jun Pan, Luosheng Wan*, Jiachun, Chen*

Hubei Key Laboratory of Natural Medicinal Chemistry and Resource Evaluation, School of Pharmacy, Huazhong University of Science and Technology, Hangkong Road 13#, Wuhan 430030, China.

Correspondence should be addressed to Jiachun Chen ([homespringchen@mail.hust.edu.cn](mailto:homespringchen@mail.hust.edu.cn)) and Luosheng Wan ([wanluosheng@hust.edu.cn](mailto:wanluosheng@hust.edu.cn)).

**Part 1. Preparation procedure of four herb extracts**

*1.1 Materials and reagents*

Coptidis Rhizoma was purchased from the *Huanglian* Production Cooperative of Jianzhuxi, Lichuan, Hubei. Liriopes Radix was purchased from the GAP planting base of *Hubei Maidong* in Oumiao Town, Xiangfan, Hubei. Fresh bitter melons were purchased from the Dianye Community Market in Jianghan District, Wuhan, Hubei. Cassiae Semen was purchased from Tongrentang Pharmacy, Jiefang Avenue, Wuhan, Hubei. Papain (12 U/mg) was purchased from Biosharp (USA). 1-pheny-3-methyl- 5-pyrazolone (PMP) was purchased from Aladdin Biochemical Technology Co., Ltd.. Ethanol, sodium chloride (NaCl), acetic acid, hydrochloric acid (HCl), methanol, potassium dihydrogen phosphate (KH_2_PO_4_), phosphoric acid (H_3_PO_4_), chloroform, trifluoroacetic acid (TFA), fructose, glucose were purchased from Sinopharm Chemical Reagent Co., Ltd. (Shanghai, China). DEAE- cellulose 52 was purchased from Whatman (UK). D101 macroporous resin was purchased from Shanghai Resin Factory Co.,Ltd. (Shanghai, China).

The mini pellicon system (Millipore, USA) was used in the ultrafiltlration (molecular weight cut-off 1000).

*1.2 Preparation of herb extracts*

*1.2.1 Preparation of total alkaloid of Coptidis Rhizoma*

Coptidis Rhizoma (1.3 kg) was ground and refluxed with 13 L 60% ethanol. The extract was concentrated in vacuum. The residue was dissolved in 1% aqueous acetic acid (5.2 L) in a water bath (55℃). The insoluble part was discarded. The pH of filtrate was adjusted to 1.0. After adding NaCl (18%, w/v), the filtrate was kept at 4℃ for 24 h and filtered. The precipitate was washed with ice water, then dried and crushed. Total alkaloid was obtained weighing 220 g.

*1.2.2 Preparation of* *Liriopes Radix polysaccharide*

Powdered Liriopes Radix (200 g) was extracted with boiled water for three times, 0.5 h each time. The ratio of crude drug and liquid was 1:4, 1:4, 1:2 (w/v), respectively. All the filtrate was collected. Phosphate buffer (pH 5.91) and 0.6 g of papain were added into the extracts and kept in a water bath (45℃) for 2 h to remove proteins. After boiled for 5 min, the extracts were stored overnight at 4 ℃ and then filtered. The filtrate was purified using the mini pellicon system with 12 L distilled water. The retentate portion was concentrated and then subjected to a DEAE-cellulose 52 column to remove pigments. Water elutant was concentrated and freeze dried. Polysaccharides were obtained weighing 60.8 g.

*1.2.3 Preparation of bitter melon extract*

Fresh bitter melons (90 kg) were squeezed, and the juice was collected. The residue was immersed with 70% ethanol overnight then squashed, and the procedure was repeated. The combined extracts were concentrated under vacuum to remove all the ethanol. This concentrated extract was subjected to alcohol precipitation with 80% ethanol for 24 h. The extract was filtered and concentrated under vacuum to remove all the ethanol, and then freeze dried. Bitter melon extract was obtained weighing 2.25 kg.

*1.2.4 Preparation of Cassia Semen extract*

Powdered Cassiae Semen (1.8 kg) was refluxed for 2 h after immersion of 80% ethanol (14.4 L) overnight. The extract was vacuum dried to remove the ethanol to yield a concentrated extract (0.6 g crude drug in 1 ml). The crude extract was subjected to D101 macroporous resin. The column was eluted with water (10 L) and then 90% ethanol (12 L). The 90% ethanol elutant was kept and vacuum dried. Cassiae Semen extract was obtained weighing 72.0 g.

**Part 2. Separation and determination of M7, M8, M10**

Fresh bitter melon (20 kg) was squeezed and the residue was extracted with 70% ethanol for twice. The bitter melon juice, along with the 70% ethanol extract, was vacuum dried and subsequently, suspended in water. This bitter melon extract was extracted with chloroform, yielding a fraction weighing 132.5 g. The CHCl_3_ fraction was then subjected to silica gel, LH-20 and finally, semi-preparative reversed-phase high performance liquid chromatography, yielding **M7 (**152 mg**)**, **M8 (**81 mg**)**, **M10** (120 mg) (**Fig. S1**).

**M7** was obtained as white powder. ^1^H-NMR (400 MHz, CD3OD) δ: 9.85, (1H, s, H-19), 5.96 (1H, br d, *J* = 5.6 Hz, H-7), 5.58 (2H, m, overlapped, H-23, 24), 4.24 (1H, d, *J* =7.8, H-1’), 1.25 (9H, s, overlapped, H-26, 27, 28), 0.94 (3H, d, *J* = 5.6 Hz, H-21), 1.08, 0.94, 0.83 (3H each, s, H-29, 18, 30), 0.95 (3H, d, *J* =5.9, H-21). ^13^C-NMR (100 MHz, CD_3_OD) δ: 210.3 (C-19), 147.9 (C-5), 140.9 (C-24), 125.8 (C-6), 123.4 (C-23), 102.1 (C-1’), 78.1 (C-5’), 78.0 (C-3’), 77.0 (C-3), 75.0 (C-2’), 73.5 (C-7), 71.7 (C-4’), 71.2 (C-25), 62.8 (C-6’), 51.3 (C-9), 51.2 (C-17), 48.8 (C-14), 46.9 (C-8), 46.6 (C-13), 42.4 (C-4), 40.2 (C-22), 37.6 (C-20), 37.4 (C-10), 35.8 (C-15), 30.1 (C-26), 30.1 (C-27), 30.0 (C-12), 29.8 (C-2), 28.4 (C-16), 27.8 (C-29), 26.0 (C-28), 23.2 (C-11), 22.3 (C-1), 19.2 (C-21), 18.8 (C-30), 15.4 (C-18). M7 was determined as momordicoside L according to previous reports [1].

**M8** was obtained as white powder. ^1^H-NMR (400 MHz, CD_3_OD) δ: 9.85, (1H, s, H-19), 5.87 (1H, br d, *J* = 5.5 Hz, H-6), 5.58 (2H, m, overlapped, H-23, 24), 4.66 (1H, d, *J* =7.8, H-1’), 4.00 (1H, br d, *J* = 5.6 Hz, H-7), 1.26 (6H, s, overlapped, H-26, 27), 0.95 (3H, d, *J* = 5.6 Hz, H-21), 1.32, 1.08, 0.92, 0.82 (3H each, s, H-28, 29, 18, 30), 0.95 (3H, d, J=5.9, H-21). ^13^C-NMR (100 MHz, CD_3_OD) δ: 210.1 (C-19), 147.8 (C-5), 140.9 (C-24), 125.8 (C-23), 123.3 (C-6), 103.8 (C-1’), 87.3 (C-3), 75.1 (C-5’), 73.2 (C-2’), 72.7 (C-3’), 71.2 (C-25), 69.0 (C-4’), 66.7 (C-7), 63.3 (C-6’), 51.4 (C-8), 51.3 (C-9), 51.1 (C-17), 49.3 (C-14), 46.6 (C-13), 42.5 (C-4), 40.3 (C-22), 37.6 (C-20), 37.3 (C-10), 35.7 (C-15), 30.1 (C-27), 30.1 (C-26), 30.0 (C-12), 28.7 (C-2), 28.5 (C-16), 27.6 (C-29), 26.1 (C-28), 23.3 (C-11), 23.0 (C-1), 19.2 (C-21), 18.7 (C-30), 15.3 (C-18). **M8** was determined as 7β,25-dihydrocucurbita-5,23(*E*)-dien- 19-al-3-O-β-D-allopyranoside according to previous reports [2].

**M10** was obtained as white powder. ^1^H-NMR (400 MHz, CDCl_3_) δ: 6.03 (br d, *J* = 9.6 Hz, H-6), 5.55 (1H each, m, overlapped, H-7, 23, 24), 4.71 (d, *J* = 7.6 Hz), 1.29 (3H each, s, H-26, 27); 0.88 (3H, s, H-29), 1.14 (3H, s, H-28), 0.86 (3H, m, overlapped), 0.84 (3H, each, s, H-18, 30). ^13^C-NMR (100 MHz, CDCl_3_) δ: 139.5 (C-24), 132.8 (C-6), 130.7 (C-7), 125.2 (C-23), 102.0 (C-1’), 86.4 (C-5), 84.2 (C-3), 79.8 (C-19), 73.9 (C-5’), 71.5 (C-2’), 70.7 (C-25), 70.6 (C-3’), 68.3 (C-4’), 63.1 (C-6’), 52.0 (C-8), 50.1 (C-17), 48.6 (C-14), 45.2 (C-13), 44.8 (C-9), 39.5 (C-10), 39.1 (C-22), 38.6 (C-4), 36.2 (C-20), 33.2 (C-15), 30.7 (C-12), 29.9 (C-27), 29.8 (C-26), 28.0 (C-16), 27.4 (C-2), 25.6 (C-29), 23.4 (C-11), 20.6 (C-28), 20.0 (C-30), 18.6 (C-21), 18.4 (C-1), 14.9 (C-18). **M10** was determined as momordicoside F_2_ according to previous reports [1].

**M7**, **M8**, **M10** was subjected to HPLC analysis to determine its purity with the same HPLC condition described in **Material and method** (**Fig. S2**).


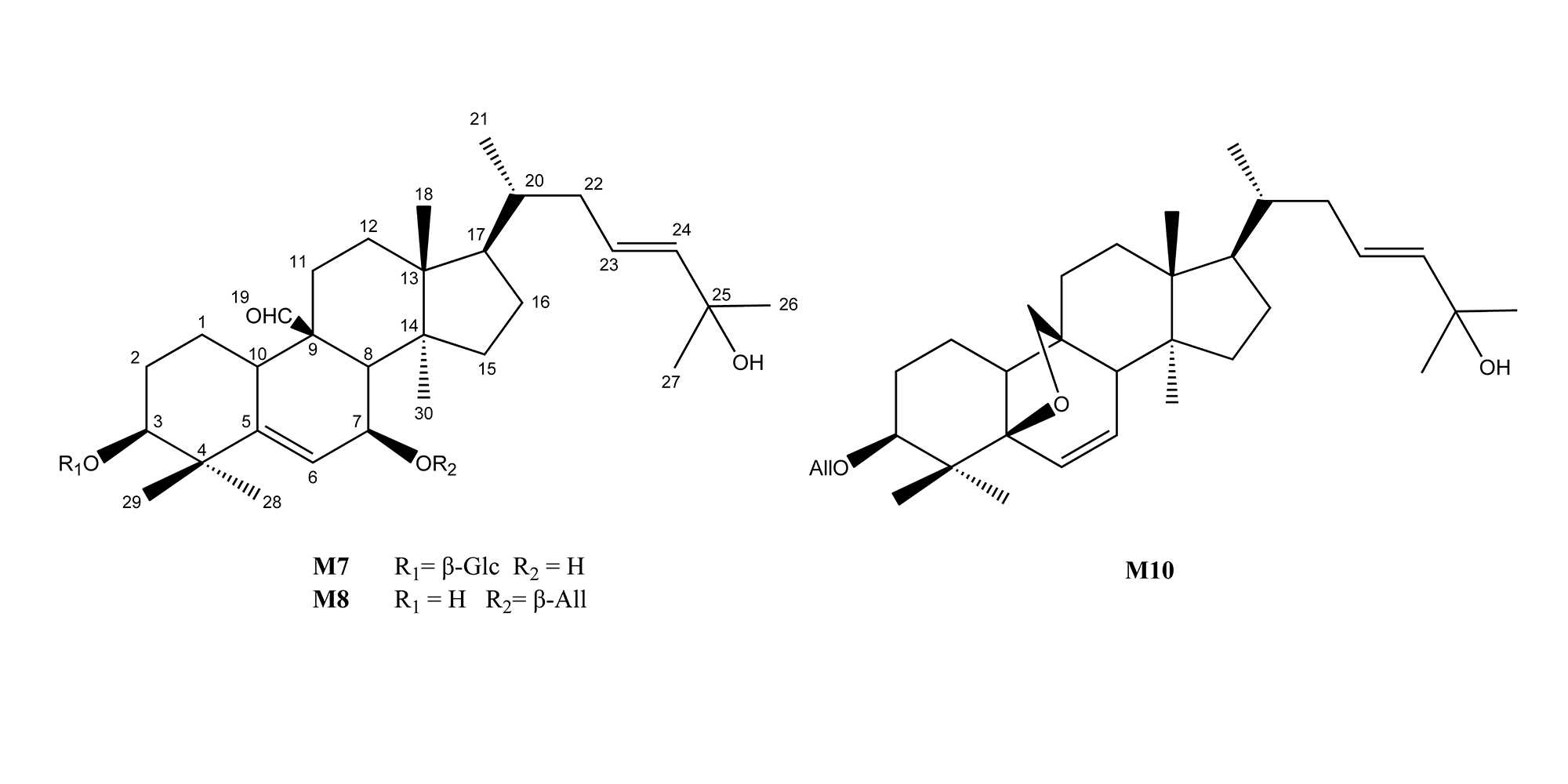


**Fig. S1**. Structures of **M7**, **M8** and **M10**.


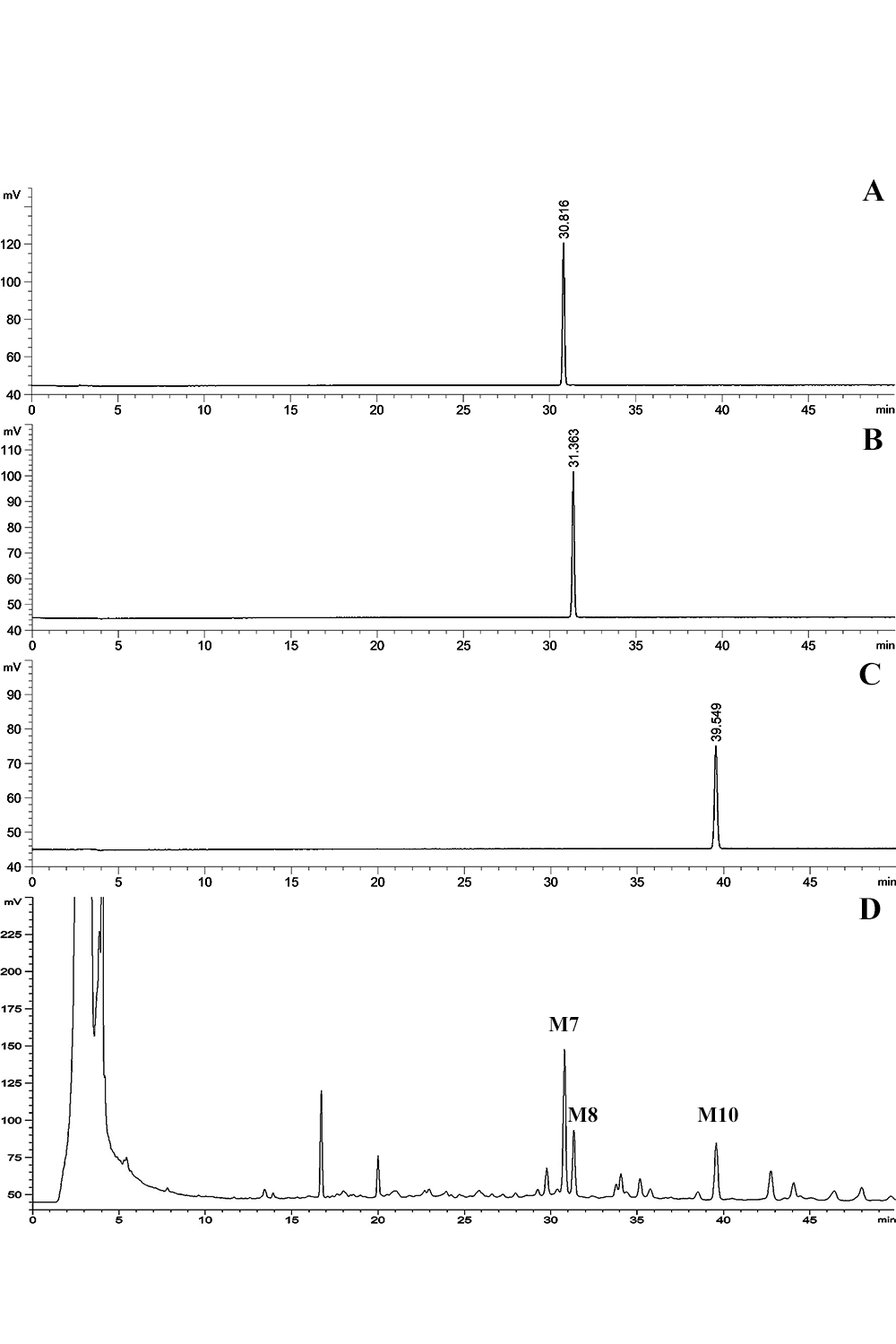


**Fig. S2**. HPLC spectra of **M7** (**A**), **M8** (**B**), **M10** (**C**) and bitter melon extract (**D**).

**Part 3. Monosaccharide composition analysis using PMP derivation**

Monosaccharide composition analysis was conducted according to previous reports with small modifications [3]. Polysaccharides (10.0 mg) were weighed accurately and dissolved in 2.0 ml water. TFA (2.0 ml) was added. The solvent was kept in a water bath (80℃) for 90 min. The reaction was terminated in ice-water bath. The reaction mixture was vacuum dried, and the residue was dissolved in a 5 ml volumetric flask. TFA treated polysaccharide solution (100 μl) were placed in a cuvette. NaOH aqueous solution (0.3 mol/L, 500 μl), PMP methanol solution (0.5 mol/L, 500 μl) were added. The reaction was then conducted in a water bath (80℃) for 4 h. Reaction liquid was then cooled under room temperature and acidified with HCl aqueous solution (0.15 mol/L, 1 ml). The solvent were extracted three times with chloroform (2 ml) to remove the PMP. The aqueous layer was sample solution. Fructose and glucose were treated likewise to yield standard solution. Sample and standard solution (20 μl) were subjected to HPLC analysis.

**Table S1 *In-Silico* Screening results of XKYS**

| ABCG2 | AURKB | CDK2 | DYRK1A | HMOX1 | KCNA7 | MMP9 | PPARG | PTGS1 | SLC6A3 |
| --- | --- | --- | --- | --- | --- | --- | --- | --- | --- |
| ACAA2 | AURKC | CDK5 | ELANE | HNF1A | KCNH2 | NCOA2 | PPARGC1A | PTGS2 | SLC6A4 |
| ACACA | AZU1 | CEBPA | ESR1 | HSD11B1 | KDM4A | NFE2L2 | PPP1CA | PTP4A3 | SOD1 |
| ACADM | BCHE | CHEK1 | ESR2 | HSD11B1L | KDM4B | NOS1 | PPP1CB | PTPN1 | SREBF1 |
| ACE | BCL2 | CHRM1 | F10 | HSP90AA1 | KDM4C | NOS2 | PPP1CC | PTPN2 | SREBF2 |
| ACHE | BCL2L1 | CHRM2 | F2 | HTR2A | KDR | NOS3 | PRKAA1 | RELA | STAT1 |
| ACOX1 | BLK | CHRM3 | F7 | HTR2B | LCK | NQO1 | PRKAA2 | RXRA | STAT2 |
| ADORA1 | CA1 | CHRM4 | FASLG | HTR2C | LEP | NR1H2 | PRKACA | SCD | STAT3 |
| ADORA3 | CA12 | CHRM5 | FASN | IKBKB | LEPR | NR1H3 | PRKCA | SCN5A | STAT4 |
| ADRA2A | CA13 | CPT1A | FLT1 | IL1B | LYN | NR3C1 | PRKCB | SIGMAR1 | SYK |
| ADRA2B | CA14 | CRYZ | FLT4 | IL4 | MAOA | NR3C2 | PRKCD | SIRT1 | TDP1 |
| ADRA2C | CA2 | CSNK2A1 | FNTA | IL6 | MAOB | PCK1 | PRKCE | SIRT3 | TFAP2A |
| ADRB2 | CA3 | CXCR3 | FNTB | INSR | MAPK1 | PDE10A | PRKCG | SLC2A1 | TNF |
| AGER | CA5A | CYP19A1 | G6PC | IRS1 | MAPK10 | PIK3CG | PRKCH | SLC2A4 | TOP2A |
| AHR | CA5B | CYP2D6 | GCG | ITGAL | MAPK14 | PIK3R1 | PRKCQ | SLC5A1 | TOP2A |
| AKR1B1 | CA7 | CYP2J2 | GCK | KCNA1 | MAPK3 | PIM1 | PRKD1 | SLC5A10 | TYR |
| AKR1B10 | CA9 | CYP7A1 | GFER | KCNA10 | MAPT | PIM2 | PRSS1 | SLC5A11 | UCP1 |
| AKR1B15 | CALM1 | DPP4 | GSK3B | KCNA2 | MBNL1 | PIM3 | PRTN3 | SLC5A2 | UCP2 |
| AKT1 | CAMK1 | DRD1 | HCK | KCNA3 | MBNL2 | PLCG1 | PTGDR | SLC5A3 | VDR |
| AMY2A | CAMKK2 | DRD2 | HMGCR | KCNA4 | MBNL3 | PNLIP | PTGER1 | SLC5A4 |  |
| AR | CASP3 | DRD3 | HMGCS2 | KCNA5 | MCP1 | PPARA | PTGER2 | SLC5A9 |  |
| AURKA | CCNA2 | DRD4 | HMOX | KCNA6 | MGAM | PPARD | PTGIR | SLC6A2 |  |

**Table S2 The gene list collected from online resources relating to T2DM**

| ABCA1 | ATF3 | CEL | FOXA2 | HLA-DQA1 | IRS4 | MAPK9 | PDPK1 | REG3A | STK11 |
| --- | --- | --- | --- | --- | --- | --- | --- | --- | --- |
| ABCB11 | ATM | CETP | FOXC2 | HLA-DQB1 | ISL1 | MBL2 | PDX1 | REN | STMN1 |
| ABCC8 | ATP10A | CFH | FOXO1 | HLA-DRB1 | ITGA2 | MC3R | PEA15 | RENBP | TACR1 |
| ABCC9 | ATP11B | CFTR | FST | HLN2 | ITGA2B | MC4R | PFKFB3 | RETN | TAS1R2 |
| ABCG1 | ATP2A3 | CHEK2 | FTO | HMGA1 | ITGAL | MC5R | PI3 | RLN1 | TAT |
| ACACA | ATP5E | CHGA | FXN | HMGCR | ITGAM | MFN2 | PIK3CA | RPL14 | TBC1D1 |
| ACACB | ATP5G1 | CLPS | G6PC | HMOX1 | ITGAX | MGAM | PIK3CB | RPS6KB1 | TBK1 |
| ACE | AVPR1A | CNDP1 | G6PC2 | HNF1A | ITGB2 | MGAT4A | PIK3CD | RRAD | TBXA2R |
| ACE2 | AXL | CNR1 | G6PD | HNF1B | ITGB3 | MGEA5 | PIK3CG | RXRA | TBXAS1 |
| ACSL4 | BACE1 | COG2 | GAD2 | HNF4A | ITLN1 | MMP2 | PIK3R1 | RXRB | TCF7L2 |
| ACTB | BAX | COMT | GAL3ST1 | HP | ITPR1 | MMP9 | PIK3R2 | RXRG | TF |
| ADAMTS9 | BCAT1 | COX1 | GANAB | HRC | JAZF1 | MSTN | PIK3R3 | RYR2 | TFAM |
| ADCY10 | BCHE | CPE | GANC | HSD11B1 | JUN | MT1A | PINK1 | S100A12 | TFAP2A |
| ADCY5 | BCL2 | CPT1A | GC | HSD11B2 | KCNA3 | MTHFR | PKLR | SAT1 | TFRC |
| ADCYAP1 | BDNF | CPT1B | GCG | HSD17B6 | KCNJ1 | MTNR1B | PKM | SCD | TG |
| ADIPOQ | BECN1 | CRAT | GCGR | HSF1 | KCNJ10 | MTOR | PLA2G2A | SCN1A | TGFBI |
| ADIPOR1 | BGLAP | CRP | GCK | HSPA1A | KCNJ11 | MTTP | PLAT | SCT | THBD |
| ADIPOR2 | BSCL2 | CRTC2 | GCKR | HSPA1B | KCNJ15 | MYC | PLCG1 | SELP | TICAM1 |
| ADORA1 | BTC | CS | GCLC | HSPA2 | KCNJ3 | MYD88 | PLIN1 | SERPINE1 | TIMP2 |
| ADORA2B | C3 | CST3 | GFPT1 | HTR1B | KCNJ5 | NAMPT | POLD3 | SFTPA1 | TLR2 |
| ADRA1A | CA2 | CTGF | GH1 | HTR2A | KCNJ6 | NEUROD1 | PON1 | SGK1 | TLR4 |
| ADRA1B | CACNA1A | CTNNB1 | GHRL | HTR2A | KCNJ9 | NEUROG3 | PON2 | SHBG | TMEM52 |
| ADRA1D | CACNA1B | CXCL5 | GHSR | HTR2B | KCNQ1 | NFATC4 | PPARA | SHC1 | TNDM |
| ADRA2A | CACNA1C | CXCR2 | GIP | HTR2C | KCTD15 | NFKB1 | PPARD | SIRT1 | TNF |
| ADRA2B | CACNA1D | CYP11B2 | GIPR | HTR7 | KIR3DL1 | NFKBIA | PPARG | SKP2 | TNFAIP3 |
| ADRA2C | CACNA1E | CYP19A1 | GJD2 | HYMAI | KLF10 | NFKBIB | PPARGC1A | SLC10A2 | TNFRSF1B |
| ADRB1 | CACNA1G | CYP2C18 | GLP1R | IAPP | KLF11 | NIDDM1 | PPARGC1B | SLC12A3 | TNFRSF25 |
| ADRB2 | CACNB3 | CYP3A4 | GNA12 | ICAM1 | KLF7 | NIDDM2 | PPP1R2 | SLC12A5 | TNFSF11 |
| ADRB3 | CALCR | DACH1 | GNB3 | IDDM2 | KRT18 | NIDDM3 | PPP1R3A | SLC13A3 | TP53 |
| AGT | CAMK1D | DDIT3 | GNPDA2 | IDE | LAD1 | NIDDM4 | PRKAA1 | SLC16A1 | TPD52 |
| AGTR1 | CAPN10 | DEDD | GORASP1 | IFNB1 | LAMP2 | NOS1AP | PRKAA2 | SLC19A2 | TRIB3 |
| AHSG | CAPN3 | DGAT1 | GPBAR1 | IFNG | LARS2 | NOS2 | PRKAB1 | SLC22A2 | TRPC6 |
| AK1 | CARTPT | DGAT2 | GPD2 | IGF1 | LBP | NOS3 | PRKAG3 | SLC22A3 | TRPM5 |
| AKR1A1 | CASP9 | DIO2 | GPR119 | IGF1R | LCN2 | NOTCH2 | PRKAR2B | SLC22A7 | TSC22D1 |
| AKR1B1 | CAT | DOK5 | GPR39 | IGF2 | LDHC | NPC1L1 | PRKCA | SLC22A8 | TSPAN8 |
| AKT1 | CAV1 | DPP4 | GPR77 | IGF2BP2 | LDLR | NPY | PRKCB | SLC27A1 | TXK |
| AKT2 | CBL | DRD1 | GPT | IGF2R | LEP | NPY2R | PRKCD | SLC2A1 | TXNIP |
| ALDH2 | CBLB | DRD2 | GPX1 | IGFBP1 | LEPR | NPY4R | PRKCE | SLC2A10 | UCP1 |
| ALMS1 | CCK | DRD5 | GRN | IGFBP2 | LGALS3 | NPY5R | PRKCZ | SLC2A2 | UCP2 |
| ALOX5AP | CCL14 | EDN1 | GSK3B | IKBKB | LIN7C | NR0B2 | PROC | SLC2A4 | UCP3 |
| ANG | CCL2 | EGR1 | GSTM1 | IL10 | LIPA | NR1H4 | PROK1 | SLC30A8 | ULK1 |
| ANXA6 | CCL5 | EIF4EBP1 | GSTT1 | IL11 | LIPC | NR1I3 | PSMA6 | SLC5A1 | USF1 |
| AOC3 | CCND2 | ENPP1 | GYS1 | IL18 | LIPE | NR3C1 | PSMD9 | SLC5A2 | UTS2 |
| AP3S1 | CCR2 | ENSA | H6PD | IL1A | LIPF | NR3C2 | PTEN | SLC9A1 | VDR |
| APOA1 | CCR5 | EP300 | HADH | IL1B | LMX1A | NR4A1 | PTGIR | SLCO4C1 | VEGFA |
| APOA2 | CD14 | EPHX2 | HBA1 | IL1RN | LPA | NR4A3 | PTGS1 | SMAD7 | VWF |
| APOA5 | CD36 | ESR1 | HBB | IL2 | LPIN1 | NRF1 | PTGS2 | SNRPB | WFS1 |
| APOB | CD38 | ESRRA | HCAR2 | IL24 | LPIN2 | OGT | PTH | SOCS1 | WNT1 |
| APOC3 | CD4 | EXT2 | HCRT | IL4 | LPL | ONECUT1 | PTK2B | SOCS2 | WNT5B |
| APOD | CD40LG | F2 | HCRTR1 | IL6 | LRP5 | OPRM1 | PTPN1 | SOCS3 | WRN |
| APOE | CD63 | F3 | HDAC1 | IL6R | LTA | PANK4 | PTPN11 | SOCS4 | XBP1 |
| APOM | CD8A | FABP2 | HERC5 | IL8 | MAFA | PARL | PTPRS | SOD1 | ZMYND8 |
| APP | CDK11A | FABP4 | HFE | ILDR2 | MAOB | PARP1 | PYGM | SOD2 | ZNHIT3 |
| APPL1 | CDK4 | FABP6 | HHEX | INPPL1 | MAP2K1 | PAX4 | RAGE | SOD3 |  |
| AQP7 | CDK5 | FAIM2 | HIF1A | INS | MAP3K5 | PBX1 | RALBP1 | SORBS1 |  |
| AR | CDKAL1 | FASLG | HIRA | INSR | MAPK1 | PCK1 | RAMP1 | SORCS1 |  |
| ARHGEF11 | CDKN1B | FBP1 | HK1 | IP6K1 | MAPK10 | PCLO | RAMP2 | SORL1 |  |
| ARHGEF12 | CDKN1C | FDPS | HK2 | IRAK1 | MAPK14 | PCSK1 | RAMP3 | SPINK1 |  |
| ARL6IP5 | CDKN2A | FFAR1 | HK3 | IRF3 | MAPK3 | PDE3B | RARRES2 | SREBF1 |  |
| ARNT | CDKN2B | FFAR2 | HKDC1 | IRS1 | MAPK8 | PDGFRB | RASD1 | SSSCA1 |  |
| ARRB2 | CEBPB | FGF21 | HLA-DOA | IRS2 | MAPK8IP1 | PDK2 | RBP4 | SST |  |

**Table S3 Information of each node in the Component-Target-Pathway network**

| **Nodes** | **Type of Nodes** | **Degree** | **Nodes** | **Type of Nodes** | **Degree** | **Nodes** | **Type of Nodes** | **Degree** |
| --- | --- | --- | --- | --- | --- | --- | --- | --- |
| M4 | Compound | 52 | ESR1 | Target | 10 | HTR2B | Target | 4 |
| Insulin Resistance | Pathway | 23 | INSR | Target | 10 | M10 | Compound | 4 |
| M3 | Compound | 23 | NOS3 | Target | 10 | M11 | Compound | 4 |
| TNF | Target | 22 | PRKCA | Target | 10 | M7 | Compound | 4 |
| Cancer Pathway | Pathway | 21 | AR | Target | 9 | MMP9 | Target | 4 |
| AKT1 | Target | 20 | G6PC | Target | 9 | SIRT1 | Target | 4 |
| AMPK Pathway | Pathway | 20 | NOS2 | Target | 9 | SOD1 | Target | 4 |
| Insulin Pathway | Pathway | 20 | PPARG | Target | 9 | ADRA2A | Target | 3 |
| PIK3CG | Target | 20 | PRKAA1 | Target | 9 | ADRA2B | Target | 3 |
| PIK3R1 | Target | 20 | PRKCB | Target | 9 | ADRA2C | Target | 3 |
| PI3K-Akt Pathway | Pathway | 19 | FASLG | Target | 8 | CCL2 | Target | 3 |
| M1 | Compound | 18 | M17 | Compound | 8 | HMGCR | Target | 3 |
| Adipocytokine Pathway | Pathway | 17 | M19 | Compound | 8 | HSD11B1 | Target | 3 |
| FoxO Pathway | Pathway | 17 | M5 | Compound | 8 | LEP | Target | 3 |
| NAFLD | Pathway | 17 | M6 | Compound | 8 | LEPR | Target | 3 |
| MAPK1 | Target | 16 | M8 | Compound | 8 | M13 | Compound | 3 |
| MAPK3 | Target | 16 | PCK1 | Target | 8 | M15 | Compound | 3 |
| M2 | Compound | 15 | SLC2A4 | Target | 8 | ACE | Target | 2 |
| MAPK10 | Target | 15 | PTGS1 | Target | 7 | ADRB2 | Target | 2 |
| MAPK14 | Target | 15 | RXRA | Target | 7 | CYP19A1 | Target | 2 |
| HIF-1 Pathway | Pathway | 14 | BCL2 | Target | 6 | DRD1 | Target | 2 |
| IKBKB | Target | 14 | F2 | Target | 6 | DRD2 | Target | 2 |
| Influenza A | Pathway | 14 | M12 | Compound | 6 | GCG | Target | 2 |
| Neurotrophin Pathway | Pathway | 14 | PLCG1 | Target | 6 | HMOX1 | Target | 2 |
| Sphingolipid Pathway | Pathway | 14 | PPARGC1A | Target | 6 | IL4 | Target | 2 |
| Chagas disease | Pathway | 13 | SLC2A1 | Target | 6 | M14 | Compound | 2 |
| Glucagon Pathway | Pathway | 13 | CPT1A | Target | 5 | M18 | Compound | 2 |
| IRS1 | Target | 13 | DPP4 | Target | 5 | NR3C1 | Target | 2 |
| PTGS2 | Target | 13 | GCK | Target | 5 | PPARD | Target | 2 |
| PTPN1 | Target | 13 | HTR2C | Target | 5 | SCD | Target | 2 |
| T2DM | Pathway | 13 | PPARA | Target | 5 | UCP2 | Target | 2 |
| Fc epsilon RI Pathway | Pathway | 12 | PRKCD | Target | 5 | CDK5 | Target | 1 |
| GSK3B | Target | 12 | PRKCE | Target | 5 | HNF1A | Target | 1 |
| TRP channels Pathway | Pathway | 12 | SREBF1 | Target | 5 | M16 | Compound | 1 |
| mTOR Pathway | Pathway | 12 | ACACA | Target | 4 | PTGIR | Target | 1 |
| M9 | Compound | 11 | ADORA1 | Target | 4 | TFAP2A | Target | 1 |
| PRKAA2 | Target | 11 | AKR1B1 | Target | 4 | VDR | Target | 1 |
| VEGF Pathway | Pathway | 11 | HTR2A | Target | 4 |  |  |  |

**Reference**

[1] H. Okabe, Y. Miyahara, T. Yamauchi, “Structures of momordicosides F_1_, F_2_, G, I, K and L, novel cucurbitacins in the fruits of *Momodica charantia* L.,” *Tetrahedron Letters*, vol.23, no.1, pp. 77–80, 1982. DOI: 10.1016/S0040-4039(00)97537-3

[2] L. Harinantenaina, M. Tanaka, S. Takaoka, M. Oda, O. Mogami, M. Uchida, Y Asakawa,. *Momordica charantia* constituents and antidiabetic screening of the isolated major compounds. *Chemical and Pharmaceutical Bulletin*, vol.37, no.52, pp. 1017-1021. 2010. DOI: 10.1002/chin.200652188

[3] J. Dai, Y. Wu, S. W. Chen, S. Zhu, H. P. Yin, M. Wang, J. Tang, “Sugar compositional determination of polysaccharides from *Dunaliella salina* by modified RP-HPLC method of precolumn derivatization with 1-phenyl-3-methyl-5-pyrazolone,” *Carbohydrate Polymers*, vol. 82, no. 3, pp. 629-635. 2010. DOI: 10.1016/j.carbpol.2010.05.029
